# Complex Parameter Landscape for a Complex Neuron Model

**DOI:** 10.1371/journal.pcbi.0020094

**Published:** 2006-07-21

**Authors:** Pablo Achard, Erik De Schutter

**Affiliations:** Theoretical Neurobiology, University of Antwerp, Belgium; University College London, United Kingdom

## Abstract

The electrical activity of a neuron is strongly dependent on the ionic channels present in its membrane. Modifying the maximal conductances from these channels can have a dramatic impact on neuron behavior. But the effect of such modifications can also be cancelled out by compensatory mechanisms among different channels. We used an evolution strategy with a fitness function based on phase-plane analysis to obtain 20 very different computational models of the cerebellar Purkinje cell. All these models produced very similar outputs to current injections, including tiny details of the complex firing pattern. These models were not completely isolated in the parameter space, but neither did they belong to a large continuum of good models that would exist if weak compensations between channels were sufficient. The parameter landscape of good models can best be described as a set of loosely connected hyperplanes. Our method is efficient in finding good models in this complex landscape. Unraveling the landscape is an important step towards the understanding of functional homeostasis of neurons.

## Synopsis

Neurons are believed to be electrical information processors. But how many models of a neuron can have similar input/output behavior? How precisely must the model parameters be tuned? These questions are crucial for models of the cerebellar Purkinje cell, a neuron with a huge dendritic arborization and a complex range of electrical outputs, for which recent experiments have demonstrated that dissimilar sets of ionic channel densities can produce similar activities. The authors have therefore used a detailed model of a Purkinje cell, released its 24 channel density parameters, and let them be optimized through an evolution strategy algorithm. They obtained 20 sets of parameters (20 models) that reproduce very precisely the original electrical waveforms. Therefore, model parameters are not uniquely identifiable. The parameters obtained vary several fold whereas small variations of these can also lead to drastically different results. Therefore, the authors have examined in more details the parameter space to gain better understanding of compensatory mechanisms in such complex models. They demonstrate that the 20 models are neither completely isolated nor fully connected, but rather, they belong to thin hyperplanes of good solutions that grid searches or random searches are likely to miss.

## Introduction

Neuronal electrical activity is governed by ion fluxes. Whereas intracellular currents are primarily determined by the cell morphology and its electrical passive properties, the major components of the electrical activity of a neuron are transmembrane currents driven by gated ionic channels present all over its surface. Small changes in the channel conductances of a neuron can lead to drastically different activities. Nevertheless, robustness of electrical activity to channel alterations, also called functional homeostasis, has recently been observed in several experiments. For example, by overexpressing the *ShaI* gene into lobster stomatogastric ganglion neurons, MacLean et al. [1] nearly doubled the expression of the transient potassium current (I_A_). This increase was spontaneously compensated by an increase of the hyperpolarization-activated current (I_h_) and the activity of the neurons remained almost unaffected. Swensen and Bean [2] have shown that similar firing patterns can be obtained in vitro from mouse Purkinje cells (PCs) with dissimilar combinations of sodium and calcium currents. The robustness of PC burst firing was also observed in mice where the expression of the sodium channel Na_v_1.6 was genetically silenced. In this case, homeostasis was maintained by an increase of calcium currents. In a recent set of experiments, Schulz et al. [3] measured potassium currents and their mRNA expression in stomatogastric crab lateral pyloric neurons and found two- to four-fold interanimal variability. They also demonstrated clear correlations in K^+^ channel expression between coupled pyloric dilatator neurons of a single crab, while a larger variation of this expression was found between crabs. Computational models made by Prinz et al. [4] and Goldman et al. [5] have demonstrated that identical network or neuron activities can be obtained from disparate modeling parameters. However, these modeling studies were limited in the number of free parameters used and in the complexity and details of the measured electrical activity. This raises the question of whether it is also possible to reproduce in full detail much more complex neuronal electrical activity with models using dissimilar sets of ionic currents.

The dendritic arborization and electrical activity of PCs are among the most complex of the brain. In this study we used the electrical activity produced by an existing model of PC [6] as the data to be reproduced by newly generated models. An evolution strategy (ES) algorithm was used to tune 24 different channel densities in these models until they reproduced the electrical activity with enough detail. We then retained 20 sets of conductances that are very different from each other, which reproduce the voltage traces of the original model in a very detailed fashion. An analysis of the parameter landscape was used to understand this diversity of parameters. Finally, a comparison with previous results or methods will demonstrate the exceptional quality of the models we found.

## Results

**Figure 1:**
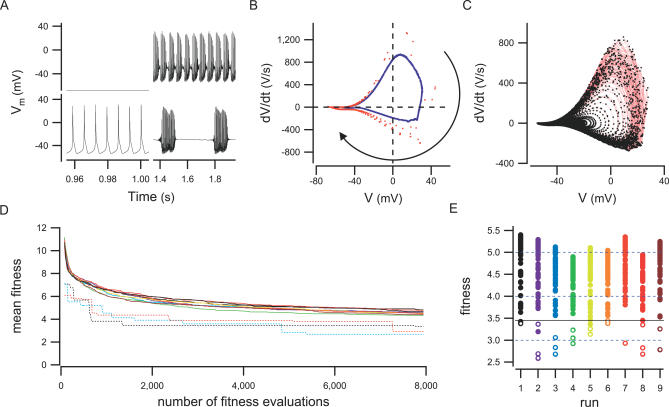
The Search Method (A) Example of the four firing modes of the PC: silent (top left), tonic (bottom left), small bursts (top right), and long bursts (bottom right) obtained with respectively 0, 0.5, 2, and 3 nA of current injected in the soma. (B) The (V,dV/dt) matrix obtained for data when a current of 0.5 nA is injected in the soma. The red points correspond to the first 0.1 s after current injection (transitory period), while the blue ones represent 1.1 s of data recorded 0.9 s after the beginning of current injection (stable period). The black arrow shows the direction followed by successive points in time during a spike. (C) The (V,dV/dt) matrix obtained for data when a current of 3 nA is injected in the soma. The black points represent 2.1 s of data recorded 0.9 s after the beginning of current injection. The red lines link successive points. (D) Time evolution of the mean fitness of the population (full lines). The nine runs, shown as different colors, have very similar evolution, and were stopped after 415 generations. The time evolution of the fitness of the best individual of runs 1, 3, and 7 is shown as dashed lines. (E) Fitness of all individuals of each population when the runs were stopped. Open points represent individuals selected for the rest of the analysis. The full line corresponds to the fitness upper limit for selecting individuals.

The original PC model, which we consider in this study as the data to be reproduced, consists of 1,600 compartments and exhibits different modes of activity depending upon the amplitude of the injected current: it can be silent, spiking, bursting with short bursts of half a dozen of spikes followed by a short inter-burst interval, or bursting with long bursts consisting of around 20 spikes and inter-burst intervals larger than 0.2 s (see Figure 1). Our generated models should reproduce not only this general behavior but also the details of electrical activity.

### Parameter Search

The cell model possesses ten different voltage or calcium-gated channels. It is subdivided in four different morphological zones with each zone exhibiting constant channel densities (see Materials and Methods). In total, there are 24 conductance parameters in this model, all of which were supposed to be unknown at the beginning of the parameter search.

Among the wide variety of optimization algorithms available, we have chosen ES. There are good theoretical and practical reasons to prefer ES to other algorithms when the parameters to tune are real numbers and their number is high [7,8]. As in other evolutionary algorithms, each set of parameters is called an “individual.” Several individuals form a “population” and populations are evolving, “generation” after “generation,” through “mutation” and “cross-over” of their individuals and “selection” of the best ones. After fine tuning of this optimization algorithm (see Protocol S1), nine runs were performed with different random number generator seeds. Each run was stopped after the evaluation of a fixed number of individuals (~8,000).

To assess the quality of an individual and to allow selection while the ES algorithm runs, a single real number, called “fitness”, measures its distance to the data. Several fitness functions have been used with electrophysiological data [9–11]. Our distance measurement is based on LeMasson's phase plane analysis [11], where the time evolution of the cell voltage, V(t), is summarized in a matrix in which V is associated with dV/dt for every time step (see Figure 1B and C). The fitness function that we have used takes into account different amplitudes of injected current, different periods in time after current injection, and different recording sites (see Materials and Methods, and Protocol S2). The fitness measure has arbitrary units and best models have lowest “fitness.” The evolution of the population fitness with each generation is shown in Figure 1D.

### Quality of the 20 Best Models

The fitnesses of the 57 individuals in each of the final nine populations are shown in Figure 1E. To check the quality of the models obtained, we compared the best models with the data by injecting current at additional amplitudes (see Figure S1). After visual examination of the best solutions, we decided to select, for the rest of the analysis, all individuals with a good fitness that exhibit the four modes of activity with somatic current injection (see Materials and Methods). The 20 good individuals that form our final selection are drawn as open circles in Figure 1E. Their fitness values are in the range 2.58 to 3.45.

**Figure 2:**
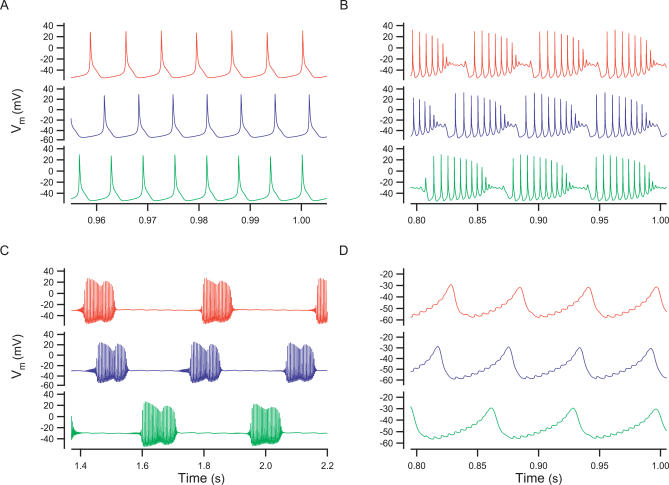
Comparison of the Models with the Data for Current Clamp (A–C) The membrane potential of the soma is shown for the data (red traces), best (blue), and worst (green) model of our selection for different somatic current amplitudes: 0.5 (A), 2 (B), and 3 nA (C). (D) Same as (B) for dendritic membrane potential.

To demonstrate the quality of this selection, the electrical activity in the soma, as well as in one dendrite, of the models with lowest and highest fitness value of the selection are shown in Figure 2 and Figure S1. All the obtained models very precisely match the data. The main macroscopic difference is due to transitions between the four modes (silence, spikes, small bursts, and long bursts) that sometimes occur at slightly different current amplitudes. The shape of the spikes, the dendritic activity, the waveform of the bursts, the activity between bursts, etc., are all reproduced accurately by every selected model (Protocol S2 and Figures S2–S4). Interestingly, the models also reproduce burst-to-burst variations that are present in the data. In Figure 2C, for example, the envelope of the bursts, as well as the length of their pre-spiking oscillations, vary both in a similar way in the data and models.

**Figure 3:**
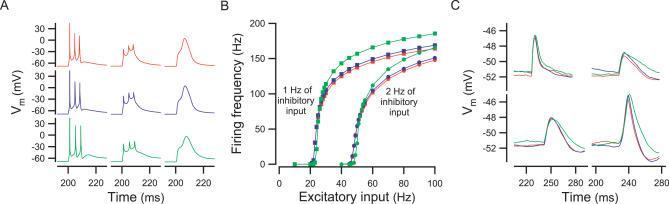
Comparison of the Models with the Data for Synaptic Responses Data (red lines), best (blue lines), and worst (green lines) model of our selection are compared for different synaptic responses. (A) Complex spike in the soma (left), main dendrite (middle), and smooth dendrite (right) after activation of the climbing fiber at time 0.2 s. (B) Simple spike frequency response to different levels of excitation and inhibition. (C) EPSPs generated by a synchronous parallel fiber input plus an asynchronous background excitation and inhibition. EPSPs are generated in four different branchlets and recorded at the soma, which is passive. The traces show the average of 40 EPSPs obtained with different random number generator seeds.

In addition, we have looked at features for which the models were not tuned, i.e. their response to synaptic input [12]. The somatic and dendritic waveforms of complex spikes generated by climbing fiber excitation are very well reproduced by all models (see Figure 3). The firing rate evoked by combined random excitatory and inhibitory input is also reproduced quite well by the best model (Figure 3B). The worst selected model has a sharper transition between the silent mode and the high frequency firing one, but it occurs at the same balance between excitation and inhibition. The active propagation of excitatory post-synaptic potentials (EPSP) [13] also remains in all models: in Figure 3C, EPSPs triggered at four different synapse locations are recorded at the soma and compared for the data and models.

All in all, these results show an exceptionally good agreement between data and models (see also Protocol S2).

### Variability of the Conductances

**Figure 4:**
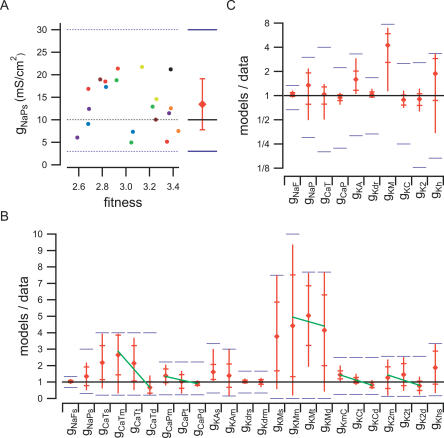
Conductance Spread (A) The fitness of each individual is plotted against its g_NaPs_ value. Points of the same color belong to the same population (see Figure 1E). Blue lines give the range in which channel densities were allowed to vary while the black line gives the value of the data. On the right, the red marker shows the mean ± sdv of the 20 values. (B) For each conductance, the mean value of the 20 selected individuals is shown, normalized to the data. The full red bars delimit the whole range covered by the 20 models while the horizontal red lines give sdv. Blue bars show the range of variation allowed during the search. Green lines are linear fit, supposing regular spacing on the abscissa. (C) Same as (B) for total conductances, obtained by summing conductance densities of the same type, weighted by the surface area of the membrane regions where they apply.

We evaluated, for each of the 24 parameters, how diverse the 20 best solutions were. As an example, the value of the maximum conductance of the persistent sodium current (g_NaPs_, see Table 1 for the list of current abbreviations) of each individual is plotted against its fitness value in Figure 4. The mean value, standard deviation (sdv), and total range of the 20 selected individuals, normalized to the data, are shown for each conductance density in Figure 4B and for summed total conductances in Figure 4C. Overall, these results exhibit large differences when compared to the data, showing that the precise reproduction of electrical activity we have shown in Figure 2 can be obtained from a wide range of parameter values. On one extreme, parameters like g_CaTm_, g_KAm_, g_KMd_, or g_Khs_ took values in the whole allowed range, while parameters like g_NaFs_, g_CaPd_, g_Kdrs,_ or g_Kdrm_ needed to be more constrained to replicate the desired activity.

**Table 1:**
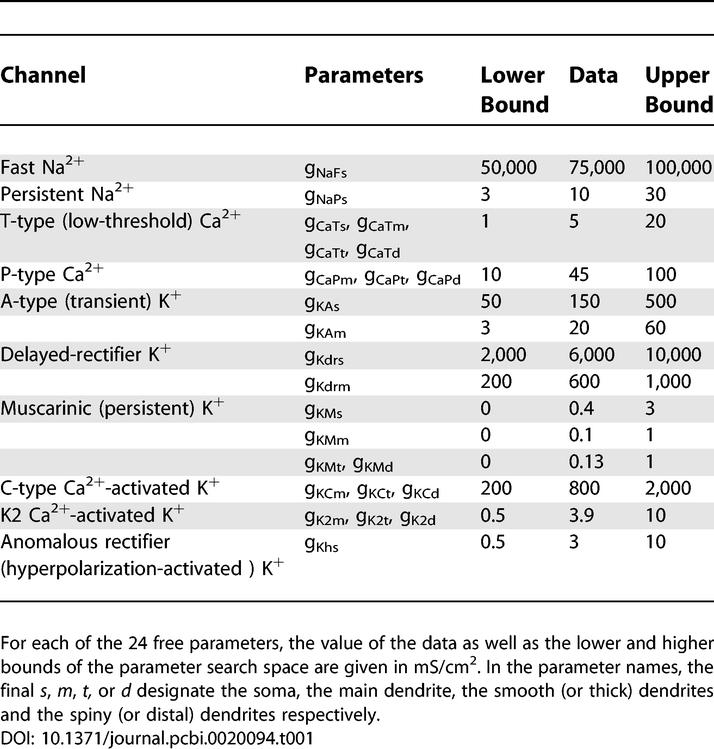
Parameter Bounds and Data Values

The spatial distribution of the conductance densities is also instructive. The decomposition of the conductances that have three dendritic components shows a clear pattern: for all of them, except g_KM_, the mean values of the 20 selected individuals are below the data in the distal spiny dendrites, while they are above it in the proximal smooth and main dendrites. This shows that using equal densities in the smooth and spiny dendrite, as was done in the original model, is not the easiest way to obtain the desired output.

What can explain this large diversity of good conductance density values? We will explore four possible hypotheses. First, the model could have too many dimensions; some parameters have a very low influence on the neuron's electrical behavior [14] and therefore they can take almost any value without prejudice. Second, all the solutions we have found could belong to a continuous region of the parameter space where the models reproduce well the data; there is a large region around the data for which changes of the parameters have small effects. Third, there could be strong compensatory mechanisms between some ionic currents, exactly as demonstrated experimentally by MacLean et al. [1] or Swensen and Bean [2]. In this case, if two currents compensate, hyperplanes of good solutions will exist in the parameter space; if more complicated correlations exist between several currents, hyperspaces of good models will be present. Fourth and opposite to the previous hypothesis, all the solutions we have found could belong to small regions of the parameter space that are isolated from each other. This would imply discontinuities such as threshold mechanisms. To disentangle these hypotheses a better knowledge of the parameter landscape is required.

### Analysis of the Parameter Landscape

**Figure 5:**
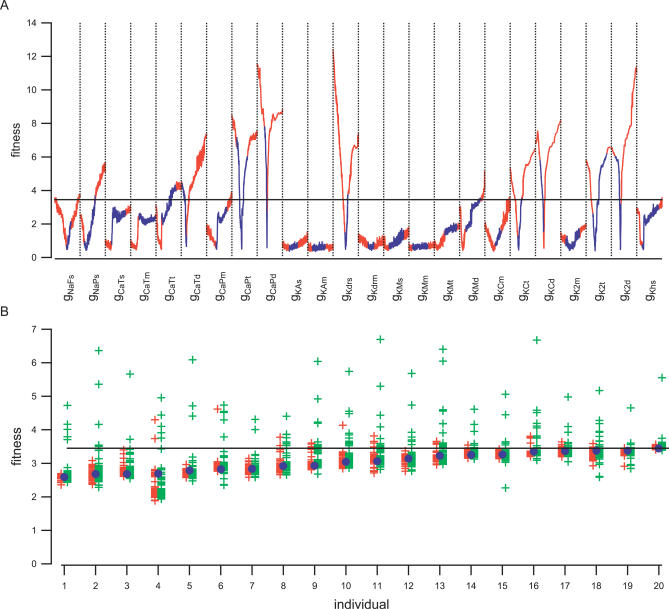
Points of the Parameter Space around the Data and the Selected Individuals (A) Fitness of 24 × 500 points for which all the parameters are equal to that of the data but one, labeled on the abscissa, and varied randomly in the full allowed range (see Table 1). The mean ± sdv range covered by the 20 selected individuals is shown in blue for each conductance density. The exact data value is never randomly selected so none of the distributions reached the perfect fitness value of 0. (B) For each of the 20 selected individuals (blue dots), the fitness of the 48 individuals obtained by changing its parameters by ±1% (red) or ±5% (green). Only one parameter is changed at a time.

We have tested how sensitive the data is to variations of each conductance separately. To do so, all the densities were set to the data value but one for which 500 random points were taken in the whole allowed interval (see Table 1). The fitness values of these 24 × 500 points are shown in Figure 5A. Seven of the parameters (g_KAs_, g_KAm_, g_Kdrm_, g_KMs_, g_kMm_, g_KMt_, g_K2m_) had a very small effect on the fitness of the models over the whole tested range. We can therefore suppose that the diversity of their values is explained by the low effect of these parameters. However, a model with these seven parameters set to zero and all other parameters equal to the data value had a poor fitness of 4.25. So, if their individual effect was small, their collective effect was still quite considerable. Additionally, since the allowed values for g_KAs_, g_KMt_, and especially g_Kdrm_ are not fully covered by our 20 models (Figure 4B), their influence was probably stronger in other locations of the parameter space.

These conclusions limit the scope of the first hypothesis, but Figure 5A is also in disfavor of the second hypothesis. As one can see, fitnesses observed in the range of parameters values corresponding to the mean ± sdv of the good solutions (blue points) are often bad. This indicates that the individuals we found can not belong to a large continuum of good solutions around the data.

Testing the third and fourth hypotheses is a bit harder, as it is impossible to try every point of the parameter space, even with a small sample of points in each dimension. Compensatory effects between two conductance densities should give, in the simplest case, linear correlations between them. Therefore we have calculated, for the 276 possible pairs of parameters, the Pearson's correlation coefficient (see Materials and Methods and Figure S5). Only five pairs of conductances had a probability of correlation (*p* < 0.01): (g_K2t_, g_K2d_), (g_Kdrm_, g_K2d_), (g_Kdrm_, g_CaPm_), (g_Kdrm_, g_K2t_), and (g_CaPt_, g_CaPd_), with respective correlation coefficients of r = −0.78, −0.63, −0.59, 0.57, and −0.58. Four of these pairs involve conductance densities that had a limited range around the data (g_Kdrm_ and g_CaPd_). Two pairs showed anti-correlation between conductances of the same channel in smooth and spiny dendrites (distinguished by the last letter *t* or *d*). But this was not true for other similar pairs, (g_CaTt_, g_CaTd_), (g_KMt_, g_KMd_) and (g_KCt_, g_KCd_), which were not correlated (*p* > 0.05). So, linear correlations could explain the large range of values found for a few parameters, but they clearly were not a general explanation for the observed variability.

The presence of the same type of ionic channels on different regions of the PC suggests that compensatory mechanisms could simply be a balance between the same currents in different locations. This would then result in a reduced dispersion of the ten total conductances of the 20 models (Figure 4C), which is indeed less pronounced than the spread of the 24 channel densities (Figure 4B). Nevertheless this dispersion is still significant: the total conductances are far from being constant. Linear correlations between pairs of total conductances were absent for 44 combinations, only the (g_CaT_, g_CaP_) pair was significantly anticorrelated with r = −0.62 (*p* < 0.01).

To test whether our 20 best models were isolated from each other in the parameter space, we varied every parameter separately by plus or minus 1% or 5% around the values of the 20 individuals. The fitnesses of these close neighbors in the parameter space are shown in Figure 5B. Several conclusions can be drawn. First, all good individuals had some good neighbors; they belonged at least to small volumes of good solutions. Second, some neighbors had a better fitness than the individuals we found, so there is room for improvement of the searching technique by applying a local optimization algorithm after the ES. Such hybrid optimization has been proven to work very efficiently [14]. Third, the parameter space was not smooth; around every good individual, a 5% difference in only one of its parameters could lead to very bad models.

**Figure 6:**
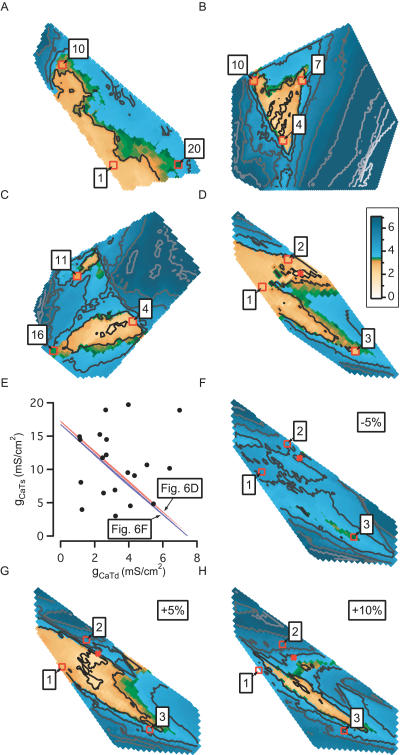
Two-Dimensional Views of Some Hyperplanes of the Parameter Space (A–D) Some typical projections onto the (g_NaPs_, g_NaFs_) plane of hyperplanes defined by triplets of individuals. The fitness values of all points belonging to these hyperplanes are color scaled. The three original individuals of each hyperplane are labeled and highlighted by a red square. Grey lines delimitate iso-fitnesses. (E) The hyperplane of (D) is shown in red in projection onto the (g_CaTs_, g_CaTd_) plane. The 20 best individuals are represented by points. A blue hyperplane is drawn parallel to the red one. It is defined by adding to the red hyperplane points 10% of the sdv of all solutions in every dimension. Note that individuals that are in between these hyperplanes in this projection can be very far away in other dimensions. (F–H) Parallel hyperplanes of (D), with the same projection. These hyperplanes are obtained by adding respectively −5%, +5%, and +10% of sdv to the points belonging to the hyperplane shown in (D). The red cross mark the region of best fitness in the original hyperplane (D).

To understand the parameter space better, we also investigated how the linear combinations of our best solutions behaved. We calculated the fitness of thousands of points in the hyperplanes defined by several triplets of solutions (see Materials and Methods). Some of these hyperplanes, projected onto the (g_NaPs_, g_NaFs_) plane, are shown in Figure 6A–6D and Supplementary Figure 6. Examples of bad models are shown in Figure S7. Regions of good models surround our 20 solutions and often link them together. But this is not always the case: in Figure 6A for example, the middle point between individuals 10 and 20 is not a good model; in Figure 6C, the individual 11 is isolated from individuals 4 and 16. None of the 20 solutions was completely isolated from others in every possible hyperplane. In Figure 6B, one can notice that some of the linear combinations of good solutions have a very good fitness value (below 2) whereas some others have a fitness comparable to that of model with completely random parameters (above 10; compare with the first points of Figure 1D).

To go further, it is interesting to visualize what happens in hyperplanes parallel to the hyperplane of Figure 6D for example. Parallel hyperplanes were defined by adding to each point of the original hyperplane −5, +5, or +10% of the sdv of the model distributions for every parameter. Two parallel hyperplanes are visible in the (g_CaTs_, g_CaTd_) plane in Figure 6E. The fitness values of points belonging to parallel hyperplanes are shown in Figure 6F−6H. With −5% of sdv (Figure 6F), almost none of the points make a good model. With +5% of sdv (Figure 6G), a band of good models still exists, but is not fully covering the best region found in Figure 6D, marked with a red cross. With +10% of sdv (Figure 6H), the region of good models is much reduced. The hypervolume delimitating good points is clearly quite restricted to the hyperplane defined by our original solutions.

### Comparison with other Methods

**Figure 7:**
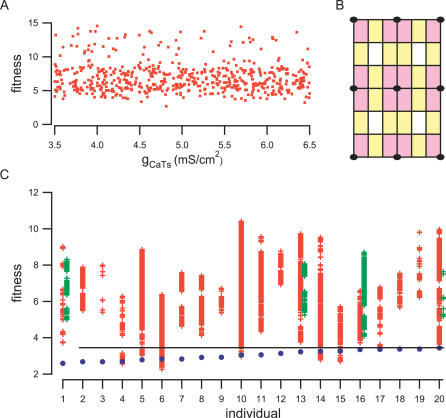
Comparison with other Methods (A) Fitness of hundreds of individuals obtained during the searching algorithm evolution, as a function of their g_CaTs_ value and centered around the data value (equal to 5). (B) Parameter space simplified to a grid of three black points in two dimensions. All individual falling in a pink region will have just one close neighbor, all the points in yellow area will have two close neighbors, and all the points in a white area will have four close neighbors. (C) Depending upon individuals (blue circles), between eight and 4,096 close neighbors are found on a six-points-per-dimension grid. The fitnesses of all these neighbors are shown as red crosses. For some individuals which have neighbors with fitness values above 3.45 but below 4 a grid of ten points per dimension was also tested (green crosses).

In Figure 7A, the fitness of hundreds of individuals obtained during algorithm evolution are shown with respect to their g_CaTs_ value. The absence of a clear relation between fitness and conductance again confirms the complex interdependencies between the different parameters of the model. Similar distributions, with as much variation, were obtained for all parameters (unpublished data). The electrical activity of the PC is a complicated combination of its conductances, making hand-tuning of parameters impossible if a very precise output is desired. In this section we investigate the likelihood that other automatic parameter search methods would discover comparable high precision models as the ES did.

Prinz et al. [15] have proposed to use systematic sampling of the parameter space as a way to tune a model's parameters. To test the performances of grid searching on our model we used a grid made of six points in every dimension. This leads to 6^24^ = 4.7 billions of billions of points to be tested which is impossible with current computing power. As we already know 20 good solutions, we looked instead at grid points which are neighbors of these solutions to see whether a grid search would have found them. Depending upon individuals, we found between eight and 4,096 close neighbors on the grid (see Materials and Methods and Figure 7B). The fitness values of these grid neighbors are shown in Figure 7C. For most of the individuals (numbered 2, 3, 7, 8, 9, 11, 12, 18, 19) all their grid neighbors had bad fitness values. It would have been impossible to find them with the grid method. Some individuals (4, 5, 6, 10, 14, 15, 17) had some grid neighbors with comparable or better fitness value and would have been discovered with the grid method. A few individuals (1, 13, 16, 20) had neighbors with fitness values above 3.45 but not too high (below 4) so their discovery might be possible with another grid resolution. To check whether this was the case, we used a grid with ten points per dimension (green markers). All the closest neighbors in a ten points grid of these individuals had very bad fitness values. So their discovery with the grid method was quite unlikely. We conclude that a grid search would have discovered only 35% of the solutions found by the ES. Of course, the ES is a stochastic process and new runs of the algorithm would certainly discover additional good individuals. Therefore the grid search is likely to find solutions that the ES did not. What we want to outline here is that, despite its enormous computing cost, a systematic sampling of the parameter space with a resolution of six or ten points per dimension fails to reveal the complexity of the parameter landscape.

## Discussion

We have shown that, by combining evolution strategies with a fitness function based on the phase-plane analysis, it was possible to replicate with high precision the electrophysiological activity of a neuron with complex firing behavior. Strengths of our search method will be described below. The large variety of conductance values found is made possible by the combined effect of several compensatory mechanisms. We have partly unraveled the very complex parameter landscape of the PC model that these mechanisms induce. The implications of these findings will be discussed.

### Strengths of the Method

Very precise models have been found in a reasonable amount of time despite the high number of variables to tune and the complexity of the cell activity. Several strong points of our method should be underlined. First of all, the phase plane method was successful in avoiding time shift problems. Indeed traces of Figure 2A were considered as almost equal by our fitness function while, because of the time shift between spikes, a fitness function based on the absolute difference between traces would give a very bad value, even worse than the one obtained by comparing a spiking to a silent neuron. The fitness function is also very simple to use as there was no need to define criteria for spiking, bursting, calcium spikes, etc., or to calculate inter spike intervals, hyperpolarizing potentials, spike widths, burst lengths, and so on, while such measures were still reproduced accurately. With neurons presenting a complex behavior, different periods of activity can be easily separated by using different matrices, as we did to distinguish the transitory period after current injection from a stable one later in time.

A second strength of the method is brought by the use of ES. As a global optimization technique, it produced results which were not local improvements of each other (compare several dots of the same color in Figure 4A). This is important to avoid falling into the local optima's trap. Unlike genetic algorithms, the parameters are coded as real numbers, so the mutation is performed by adding a random number generated from a Gaussian distribution. The width of this distribution can evolve with generations and even be part of the genotype of the individuals. This kind of evolution is impossible with genetic algorithms, where only the probability of a mutation to occur, but not its strength, can be varied. Also, the standard crossover approaches in genetic algorithms request that correlated parameters are put side-by-side in the genotype, which is difficult to achieve when the correlations between parameters are poorly understood. No such requirement exists with ES.

A couple of interesting conclusions can be made about the original PC model if we consider it as a solution among others to reproduce experimental data. The behavior of the 20 models with respect to the synaptic responses demonstrated that the original model was quite robust in this regard. This is important since, at the time this model was made, these synaptic responses were predictions or tests of its goodness [12,13]. Oppositely, the spatial distribution of conductances in the 20 selected models shows that the original choice, driven by simplicity, of equalizing smooth dendritic and spiny dendritic channel densities was not the best choice for reproducing the desired output.

The quality of the models we obtained can be compared with similar studies published earlier. On one hand, several groups have tested evolutionary algorithms. However, they were trying to tune much simpler cell models. For example, the neocortical pyramidal neuron used by Keren et al. [9] was either silent or fired tonically without much adaptation. That was also the case for the three active models tuned by Vanier and Bower [10].

On the other hand, the grid method was applied by Goldman et al. [5], as well as Prinz et al. [4], for tuning the parameters of a single neuron or a small network. These models showed more complex activity but had only a small number of free parameters, which can hardly be augmented for computing time reasons. Additionally, in certain cases, the high number of models evaluated made it impossible, for memory concerns, to save all the membrane potential values. So, while lobster stomatogastric neuron models studied by Prinz et al. [15] were correctly reproducing data for the characteristics that were explicitly measured (periodicity, burst duration, duty cycle, phase-response properties), the actual voltage traces were quite different when studied in more detail (see their Figure 9C–9D). The grid method may provide a good description of the parameter landscape of simple models but the parameter landscape of the PC model was too complex for this method (Figure 7C).

We have to notice however that the goodness of these results depends on some assumptions about the available data. First the morphology of the cell needs to be already known. While technically reconstruction of real cell morphologies poses little problems, great variability relevant for the properties of models has been observed between different laboratories [16]. Second, the kinetics of the channels were not modified. We assumed, like for the original PC model [12], that the kinetic parameters were sufficiently constrained to have biologically plausible highly precise models.

### Precise Replication of Neuronal Activity from Different Sets of Conductances

Several very good solutions were found, each comprising a different set of parameter values. The observed differences between the good models are not insignificant, since variations of the same magnitude, applied to each parameter separately, resulted in bad models (Figure 5A). This observation is in agreement with what was expected from the experimental data. The possibility to have the same behavior for computational models from different sets of conductances was already shown by Goldman et al. [5], as well as by Prinz et al [4]. But this is, to our knowledge, the first time that very different solutions were found to reproduce much more complex neuronal activity in such detail.

The required level of detail influences the ratio of maximal conductances of identically behaving models. Indeed, in this study, this ratio ranges for total conductances between 1.2 for g_NaF_ to 6.2 for g_KM_ (see Figure S8) and 9.7 for g_Kh_, but we have shown that this latter current has a small influence on the PC model. In stomatogastric ganglion neuronal models [4,5], this ratio is one order of magnitude higher: maximal conductances can vary by factors up to 40 fold. But experimentally measured variations in crab stomatogastric ganglion neurons [3] are 2–4 fold, much closer to our findings.

Altogether, recent experimental and theoretical studies are changing our view on how neurons' electrical activity is shaped. For a long time, ionic channel expression was assumed to be relatively constant, hard coded in the genome of the cell and therefore allowing easy classification of neuron types. We have come to realize that instead, channel expression [17] is constantly being regulated to obtain the desired electrical activity. The modulation state of existing channels could be regulated as well, for example, by cAMP or intracellular calcium dynamics. The mechanisms of this functional homeostasis have still to be deciphered and we believe that further studies of parameter landscapes of different neuron models will provide important contributions.

Our analysis of the PC parameter landscape demonstrate that several mechanisms contribute to the large differences between the good models. First, some channels had very small effects on the electrical behavior of the model. Second, a few conductance densities were linearly correlated or anti-correlated. Anti-correlation of calcium channel total conductances probably implies that the total amount of calcium that flows in and out of the cell is severely constrained. Anti-correlation of some ionic channels between different regions of the dendrites was also observed. Third, compensation mechanisms between different ionic currents caused small continuous regions of good models in the parameter pace. These regions were quite limited to planes intersecting some good individuals. They did not fully cover the hyperplanes of averages, indicating that threshold mechanisms or non-linear correlations between currents made some averages of good solutions behave badly, as has been reported previously in simpler models [18]. Understanding how these different mechanisms act together to maintain a precise output and discovering their molecular basis remain two fascinating challenges.

## Materials and Methods

### The cell model.

The model of PC we use has been fully described as “PM9” [6]. The ionic channels are distributed in the four different physiological zones as follows: the soma has Na_F_, Na_P_, Ca_T_, K_dr_, K_h_, K_M,_ and K_A_ conductances, whereas the dendrites have Ca_P_, Ca_T_, K_M_, K_C_ and K_2_ conductances. In addition, the main dendrite expresses also K_dr_ and K_A_ channels. This leads to a total of 24 channel density parameters to be tuned. They were allowed to vary within a wide physiological range. The bounds for each parameter, as well as the values of the data, are given in Table 1. The synaptic properties are exactly the same for the data and the models and are taken from [12].

### The search algorithm.

We have used an evolutionary algorithm belonging to the family of ES. The strategy used in the different runs is called (57+19), meaning that, at each generation, the population is composed of 57 individuals and produces 19 offspring. Members of the next generation are selected among the 57 parents, plus 19 children, according to their fitness value. The mutation parameters are self-adaptive and correlated: the width of Gaussian mutations are included in the chromosome and evolve with generations, as well as their covariance matrix angles (see [7] for details). The recombination scheme used is called “global intermediary”: two parents are selected for each parameter and the offspring inherits the average values for their parameter.

The PC model was simulated with Genesis 2.2.1 software [19] under Mac OS X. A ready-to-use ES C++ implementation, called ESEA, is available within the free Evolving Objects library [20]. We have used a parallel version of it on a cluster of ten Apple 2.3 GHz G5 dual processor nodes. Each run comprising 415 generations took around 6 d of computing.

### The fitness function.

In LeMasson's phase plane analysis [11], the fitness measurement is based on the (V, dV/dt) matrix. The electrophysiological trace V(t) is sampled with a frequency of 50 kHz. Each point has V and a dV/dt value and can therefore be ordered in a two-dimensional matrix. The distance between data and model is then given by the difference in density for every cell of the matrix:


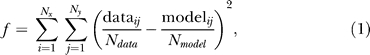


where data*_ij_* and model*_ij_* represent the number of points in the matrix cell (*i,j*) for data and model respectively; *N_data_* and *N_model_*, the total number of data and model points; and *N_x_* and *N_y_*, the size of the matrix. We have chosen 100 × 100 matrices, where V can vary between −80 and +120 mV and dV/dt between −1500 and +2500 mV/ms.

The fitness function *F* that we have used is a weighted sum of such *f*:


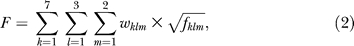


where *k* tags different injected current amplitudes: 1.5 nA injected in one thick dendrite, no current, 0.5, 1, 1.5, 2.5, and 3 nA injected in the soma; *l* corresponds to different recording sites: the soma and two thick dendrites; *m* addresses different time periods: the first 100 ms after current injection (the “transitory period”, giving red points in Figure 1B) or the entire recording after 1s (0.5 s if no current is injected) after the beginning of the experiment (the “stable period” giving blue points in Figure 1B). The weights *w_klm_* were equal to 1 when *l* designates soma recordings and 0.5 for dendritic recordings, except for null current injection where they are equal to 0.6 and 0.3 respectively.

### The selection of best individuals.

To check the quality of the models obtained in nine runs of the optimization algorithm, we have simulated the best models with 15 different injected current amplitudes: 1.5 nA in the dendrite, no current, from 0.25 nA to 3 nA with steps of 0.25 nA in the soma and 4 nA in the soma. We have examined all the best solutions and decided to select, for the rest of the analysis, individuals with a fitness below 3.45. Among the 23 individuals that fulfilled this criterion, three were rejected from our final selection because they were bursting and not spiking at the lowest injected current in the soma (0.25 nA). We are certain that these individuals were able to fire in a spiking mode at lower current amplitudes since they were spiking when current was injected in the dendrite. Nevertheless, we preferred not to include them in the rest of the analysis.

### Visualization of hyperplanes.

From several triplets of solutions, we defined hyperplanes as follows: every point of it was a weighted sum of the three solutions, the first two weights vary from −1.5 to 2.5 by step of 0.04 and the third weight is such that the sum of the weights is equal to 1. All the points with some negative parameters were rejected, which caused multiple borders to the hyperplanes. In total we had thousands of points for each hyperplane. We ran simulations for every point and computed its fitness. In the Figure 6 and Figure S6, the “land and sea” color scale allows a clear distinction between models above and below the 3.45 value that we used as a threshold between good and bad models. Grey lines in the figures link points that have the same integer fitness values.

### Grid neighbors selection.

We have used a grid of six (and later ten) points in 24 dimensions. For each dimension, an individual is located between two grid points. If the distance to the closest point was < 1/3 of the distance between two grid points, we considered only the closest neighboring point in this dimension. If not, we considered both. As an illustrative example in the Figure 7B, the grid is simplified to three (black) points in two dimensions. All individuals falling in a pink region will have just one close neighbor, all the individuals in yellow areas will have two close neighbors, and all the individuals in white areas will have four close neighbors (two in each dimension).

### Data analysis.

Data analysis was done with Igor Pro 5.04b software. The linear correlations between conductances were tested by a simple Pearson's correlation. To obtain total conductances we summed channel densities, normalized to the membrane area where they apply.

## Supporting Information

Figure S1 Full Comparison of Models with Data (A) Comparison of somatic voltages between data (red), the best (blue), and the worst (green) model with 15 injected currents. The amplitudes of injected current are labeled on the left axis where “rest” means no current injected, “d 1.5 nA” means 1.5 nA injected in the dendrite and “s xx nA” means xx nA injected in the soma. Amplitudes included in the fitness function are in bold fonts. The voltage scale is the same for all traces (see Figure 2 for scales).
(B) Same as (A). During 30 ms (spiking traces), 200 ms (small bursting traces), or 800 ms (long bursting traces) only.
(C) Same as (B) for dendritic voltage. The voltage scale is larger than in (A) and (B) but the same for all traces in (C).
(2.9 MB PDF).

Figure S2 Data/Model Comparison for Spike Parameters for 0.25-nA Current Injected Protocol The data (mean ± sdv shown as black horizontal bars) is compared to the 20 selected models (means shown as red circles and sdv as vertical bars) for:
(A) the spike duration; (B) the time between the start and the peak of the spikes; (C) the duration of after-hyperpolarizing potentials; (D) the full width at half maximum of the spikes; (E) the inter-spike interval; (F) the spike area; (G) the spike amplitude; (H) the after-hyperpolarizing potentials amplitude; (I) the potential at which spikes start; (J) the potential at half maximum of the spikes; (K) the potential of the spike peaks; and, (L) the after-hyperpolarizing potentials. See Figure 2A for typical electrophysiological traces. Variability observed in real electrophysiological data is usually of a couple of mV for the spike height and a few tenths of ms for the spike width.
(883 KB PDF)

Figure S3 Data/Model Comparison for Spike Parameters for 3-nA Current Injected Protocol Same as Figure S2 during bursting behavior with 3 nA injected current. See Figure 2C for typical electrophysiological traces.
(893 KB PDF)

Figure S4 Data/Model Comparison for Burst Parameters for 3-nA Current Injected Protocol The data (mean ± sdv shown as black horizontal bars) is compared to the 20 selected models (means shown as red circles and sdv as vertical bars) for:
(A) the inter-burst interval; (B) the duration of bursts; (C) the number of spikes per burst; and, (D) the inter-spike interval inside bursts.
(594 KB PDF)

Figure S5 Linear Correlation between Conductance Densities (A) For each pair of conductance densities a Pearson's correlation coefficient is calculated. five couples have a probability *(p)* to have a null correlation below 1%.
(B) Distribution of g_K2t_ vs. g_K2d_ for the 20 selected individuals. The colors of the points are the same as in Figure 1E. The axes extend over the limits in which conductance densities were allowed to vary. The black cross indicates the value of the data.
(C–F) Same as (B) for other pairs of correlated conductance densities.
(716 KB PDF)

Figure S6 Examples of other Hyperplanes Same as Figure 6A–6D.
Red crosses in Figure S6B label models that are shown in Figure S7.
(2.9 MB PDF)

Figure S7 Examples of Badly Behaving Models Electrophysiological activity of the three models marked with a red cross in Figure S6B. The models have a fitness equal to 3.9997 (red), 5.0003 (green), and 6.0001 (blue). The abscissa and ordinates are the same as in Figure S1.
(3.0 MB PDF)

Figure S8 Range of Variation of the Total Conductances Ratio of maximal over minimal value found for the total conductances of Figure 4C.
(18 KB EPS)

Protocol S1 Fine Tuning of the Search Method (24 KB DOC)

Protocol S2 Accuracy of the Models (28 KB DOC)

## Abbreviations

ES: evolution strategy
EPSP: excitatory post synaptic potentials
g^XX^: maximum conductance of a current XX
I_A_: transient potassium current
I_h_: hyperpolarization-activated current
PC: Purkinje cell
sdv: standard deviation
V(t): time evolution of the cell voltage

## References

[pcbi-0020094-b001] MacLean JN, Zhang Y, Johnson BR, Harris-Warrick RM (2003). Activity-independent homeostasis in rhythmically active neurons. Neuron.

[pcbi-0020094-b002] Swensen AM, Bean BP (2005). Robustness of burst firing in dissociated Purkinje neurons with acute or long-term reductions in sodium conductance. J Neurosci.

[pcbi-0020094-b003] Schulz DJ, Goaillard JM, Marder E (2006). Variable channel expression in identified single and electrically coupled neurons in different animals. Nat Neurosci.

[pcbi-0020094-b004] Prinz AA, Bucher D, Marder E (2004). Similar network activity from disparate circuit parameters. Nat Neurosci.

[pcbi-0020094-b005] Goldman MS, Golowasch J, Marder E, Abbott LF (2001). Global structure, robustness, and modulation of neuronal models. J. Neurosci.

[pcbi-0020094-b006] De Schutter E, Bower JM (1994). An active membrane model of the cerebellar Purkinje cell. I. Simulation of current clamps in slice. J Neurophysiol.

[pcbi-0020094-b007] Eiben AE, Smith JE (2003). Introduction to Evolutionary Computing.

[pcbi-0020094-b008] De Schutter E, Ekeberg O, Kotaleski JH, Achard P, Lansner A (2005). Biophysically detailed modeling of microcircuits and beyond. Trends Neurosci.

[pcbi-0020094-b009] Keren N, Peled N, Korngreen A (2005). Constraining compartmental models using multiple voltage recordings and genetic algorithms. J Neurophysiol.

[pcbi-0020094-b010] Vanier MC, Bower JM (1999). A comparative survey of automated parameter-search methods for compartmental neural models. J Comput Neurosci.

[pcbi-0020094-b011] LeMasson G, Maex R, De Schutter E (2001). Introduction to equation solving and parameter fitting. Computational neuroscience: Realistic modeling for experimentalists.

[pcbi-0020094-b012] De Schutter E, Bower JM (1994). An active membrane model of the cerebellar Purkinje cell. II. Simulation of synaptic responses. J Neurophysiol.

[pcbi-0020094-b013] De Schutter E, Bower JM (1994). Simulated responses of cerebellar Purkinje cells are independent of the dendritic location of granule cell synaptic inputs. Proc Natl Acad Sci U.S.A.

[pcbi-0020094-b014] Rodriguez-Fernandez M, Mendes P, Banga JR (2006). A hybrid approach for efficient and robust parameter estimation in biochemical pathways. BioSystems.

[pcbi-0020094-b015] Prinz AA, Billimoria CP, Marder E (2003). Alternative to hand-tuning conductance-based models: Construction and analysis of databases of model neurons. J Neurophysiol.

[pcbi-0020094-b016] Szilágyi T, De Schutter E (2004). Effects of variability in anatomical reconstruction techniques on models of synaptic integration by dendrites: A comparison of three internet archives. Eur J Neurosci.

[pcbi-0020094-b017] Toledo-Rodriguez M, Blumenfeld B, Wu C, Luo J, Attali B (2004). Correlation maps allow neuronal electrical properties to be predicted from single-cell gene expression profiles in rat neocortex. Cereb Cortex.

[pcbi-0020094-b018] Golowasch J, Goldman MS, Abbott LF, Marder E (2002). Failure of averaging in the construction of a conductance-based neuron model. J Neurophysiol.

[pcbi-0020094-b019] Bower JM, Beeman D (1998). The book of GENESIS: Exploring realistic neural models with the GEneral NEural SImulation System, 2nd Edition.

[pcbi-0020094-b020] Keijzer M, Merelo JJ, Romero G, Schoenauer M, Collet P, Fonlupt C, Hao JK, Lutton E, Schoenauer M (2001). Evolving objects: A general purpose evolutionary computation library. Artificial Evolution : 5th International Conference, Evolution Artificielle, EA 2001, Le Creusot, France, October 29–31, 2001.

