# Supplementary material for: Complex Parameter Landscape for a Complex Neuron Model
Source: PLoS Comput Biol. 2006 Jul 21;2(7):e94. doi: 10.1371/journal.pcbi.0020094 (PMC1513272; doi:10.1371/journal.pcbi.0020094)
Supplement: Supplementary file 2 [file pcbi.0020094.sg002.pdf]

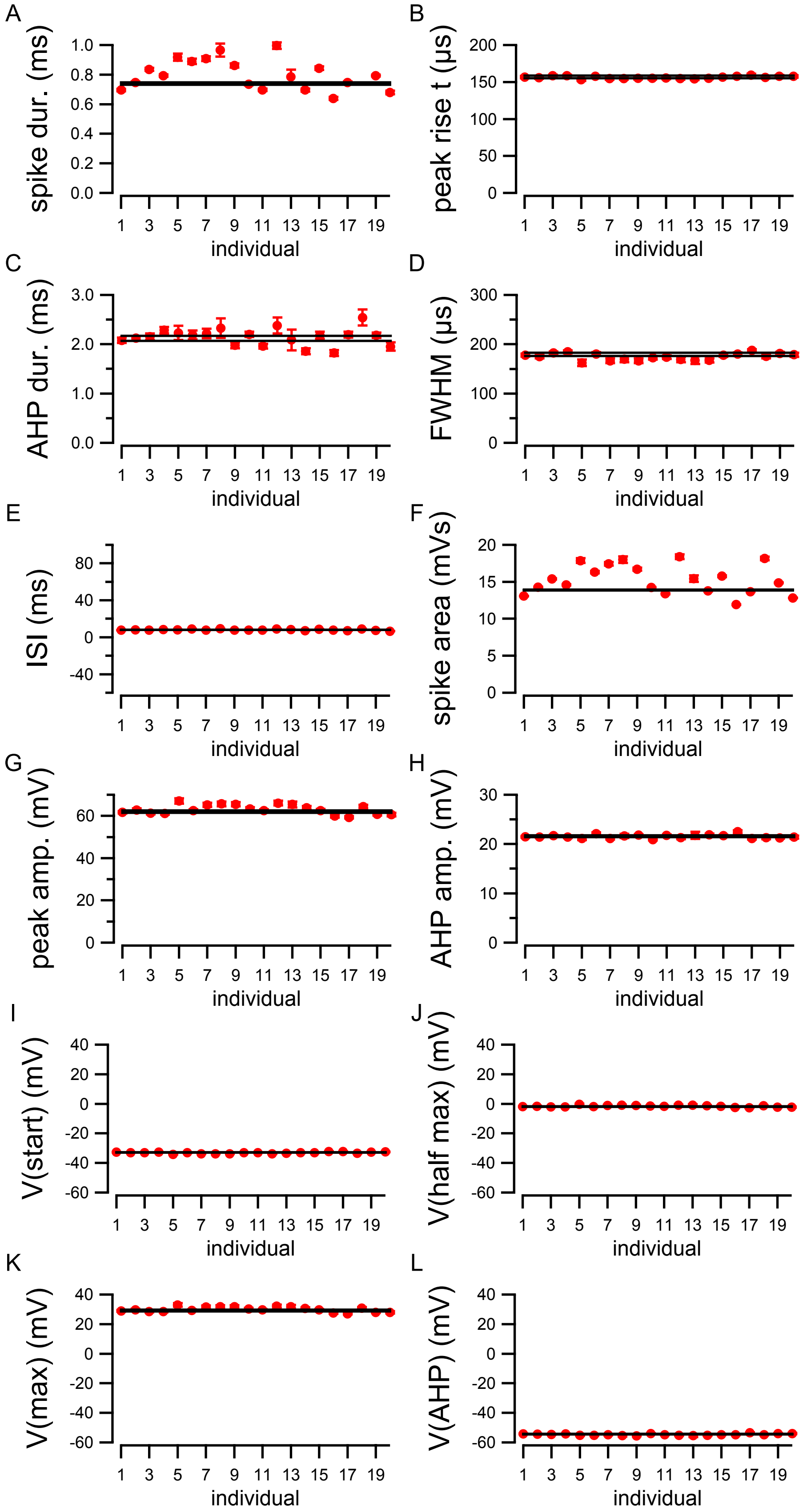
A
spike dur. (ms)
individual
B
peak rise t (µs)
individual
C
AHP dur. (ms)
individual
D
FWHM (µs)
individual
E
ISI (ms)
individual
F
spike area (mVs)
individual
G
peak amp. (mV)
individual
H
AHP amp. (mV)
individual
I
V(start) (mV)
individual
J
V(half max) (mV)
individual
K
V(max) (mV)
individual
L
V(AHP) (mV)
individual
