# Supplementary material for: Complex Parameter Landscape for a Complex Neuron Model
Source: PLoS Comput Biol. 2006 Jul 21;2(7):e94. doi: 10.1371/journal.pcbi.0020094 (PMC1513272; doi:10.1371/journal.pcbi.0020094)
Supplement: Supplementary file 3 [file pcbi.0020094.sg003.pdf]

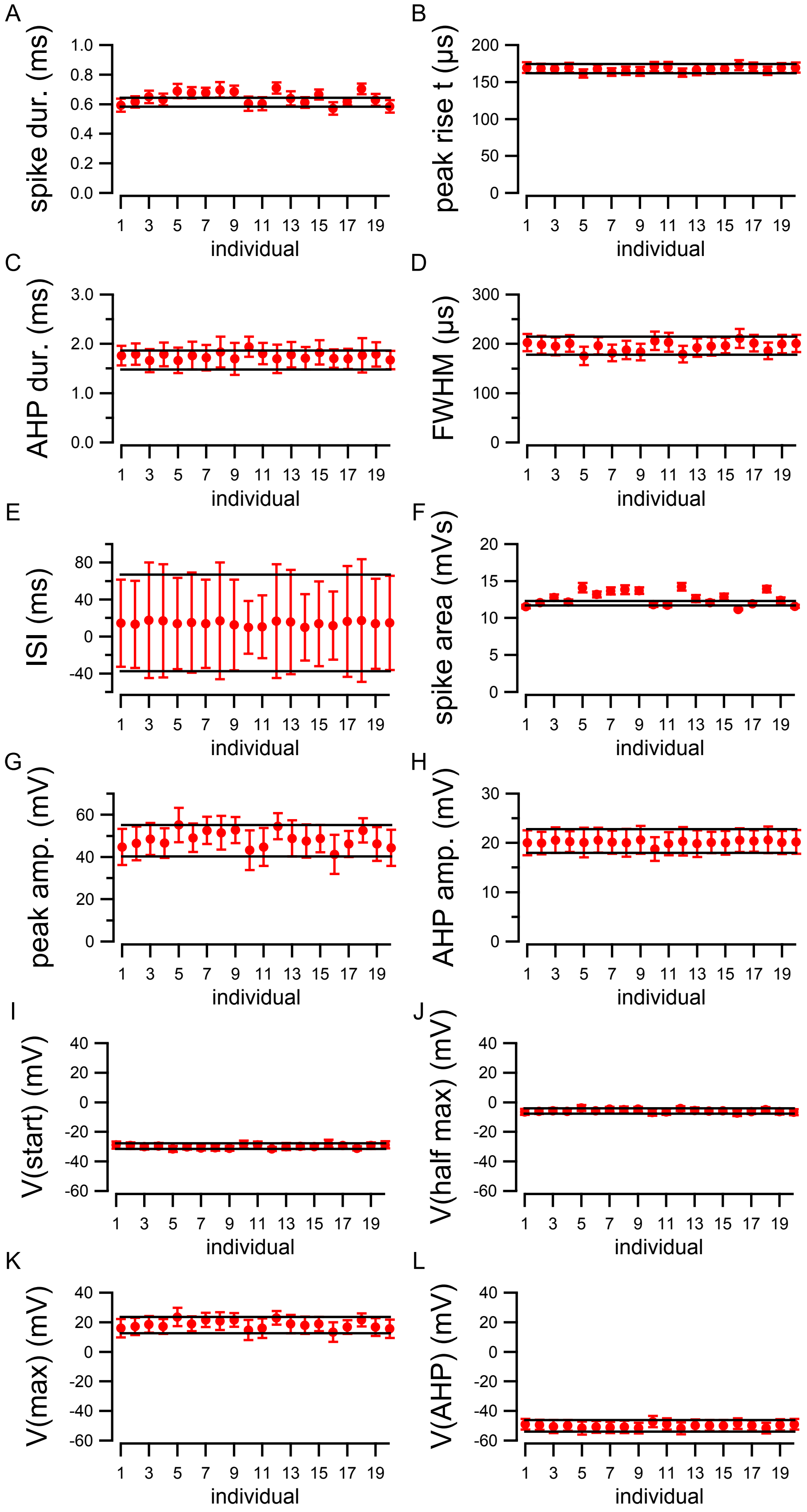
A
spike dur. (ms)
1.0
0.8
0.6
0.4
0.2
0.0
B
peak rise t (µs)
200
150
100
50
0
C
AHP dur. (ms)
3.0
2.0
1.0
0.0
D
FWHM (µs)
300
200
100
0
E
ISI (ms)
80
40
0
-40
F
spike area (mVs)
20
15
10
5
0
G
peak amp. (mV)
60
40
20
0
H
AHP amp. (mV)
30
20
10
0
I
V(start) (mV)
J
V(half max) (mV)
K
V(max) (mV)
L
V(AHP) (mV)
40
20
0
-20
-40
-60
1
3
5
7
9
11
13
15
17
19
individual
