# Supplementary material for: Complex Parameter Landscape for a Complex Neuron Model
Source: PLoS Comput Biol. 2006 Jul 21;2(7):e94. doi: 10.1371/journal.pcbi.0020094 (PMC1513272; doi:10.1371/journal.pcbi.0020094)
Supplement: Supplementary file 4 [file pcbi.0020094.sg004.pdf]

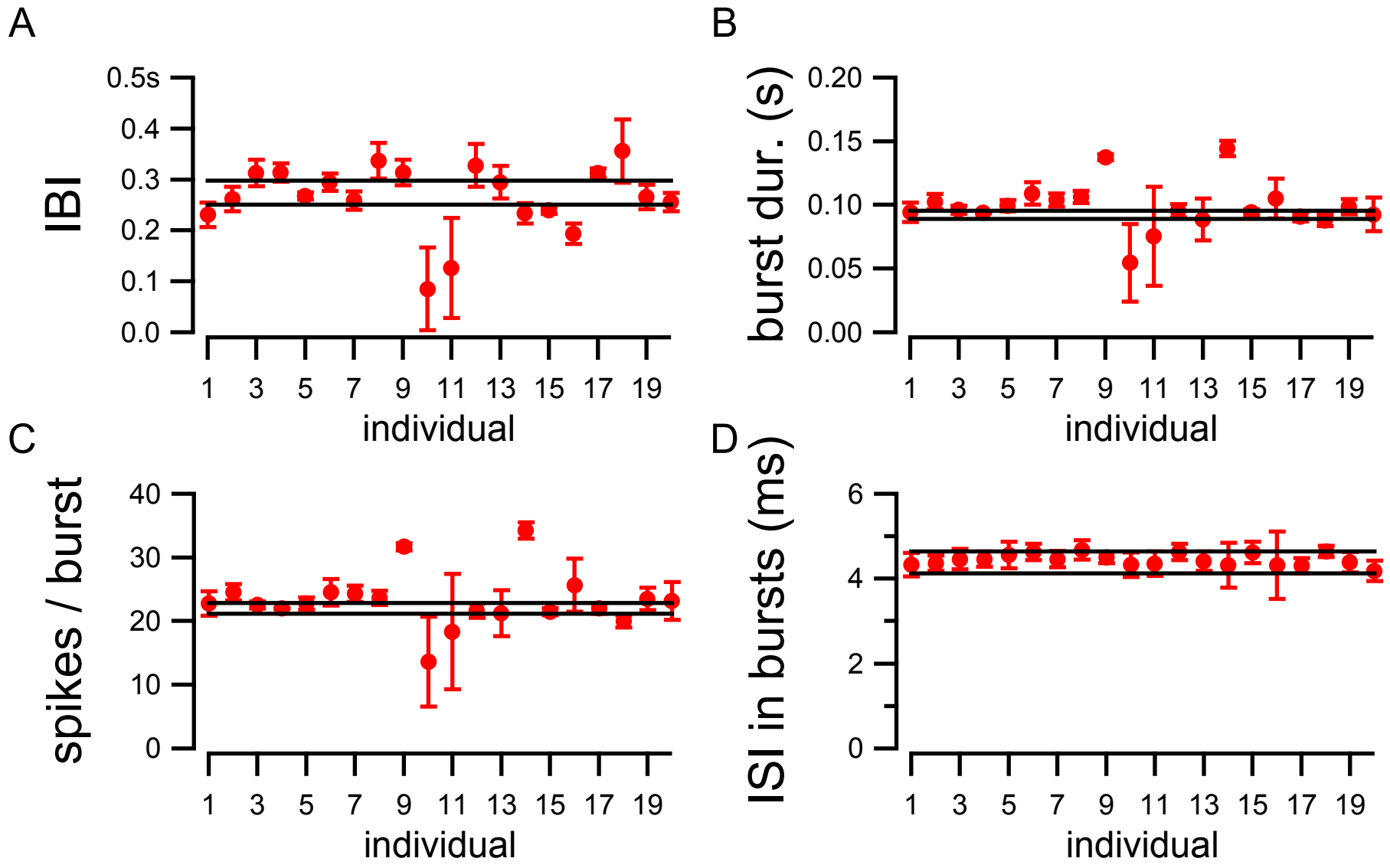

A
IBI
0.5s
0.4
0.3
0.2
0.1
0.0
1 3 5 7 9 11 13 15 17 19
individual
B
burst dur. (s)
0.20
0.15
0.10
0.05
0.00
1 3 5 7 9 11 13 15 17 19
individual
C
spikes / burst
40
30
20
10
0
1 3 5 7 9 11 13 15 17 19
individual
D
ISI in bursts (ms)
6
4
2
0
1 3 5 7 9 11 13 15 17 19
individual
