# Supplementary material for: Complex Parameter Landscape for a Complex Neuron Model
Source: PLoS Comput Biol. 2006 Jul 21;2(7):e94. doi: 10.1371/journal.pcbi.0020094 (PMC1513272; doi:10.1371/journal.pcbi.0020094)
Supplement: Supplementary file 5 [file pcbi.0020094.sg005.pdf]

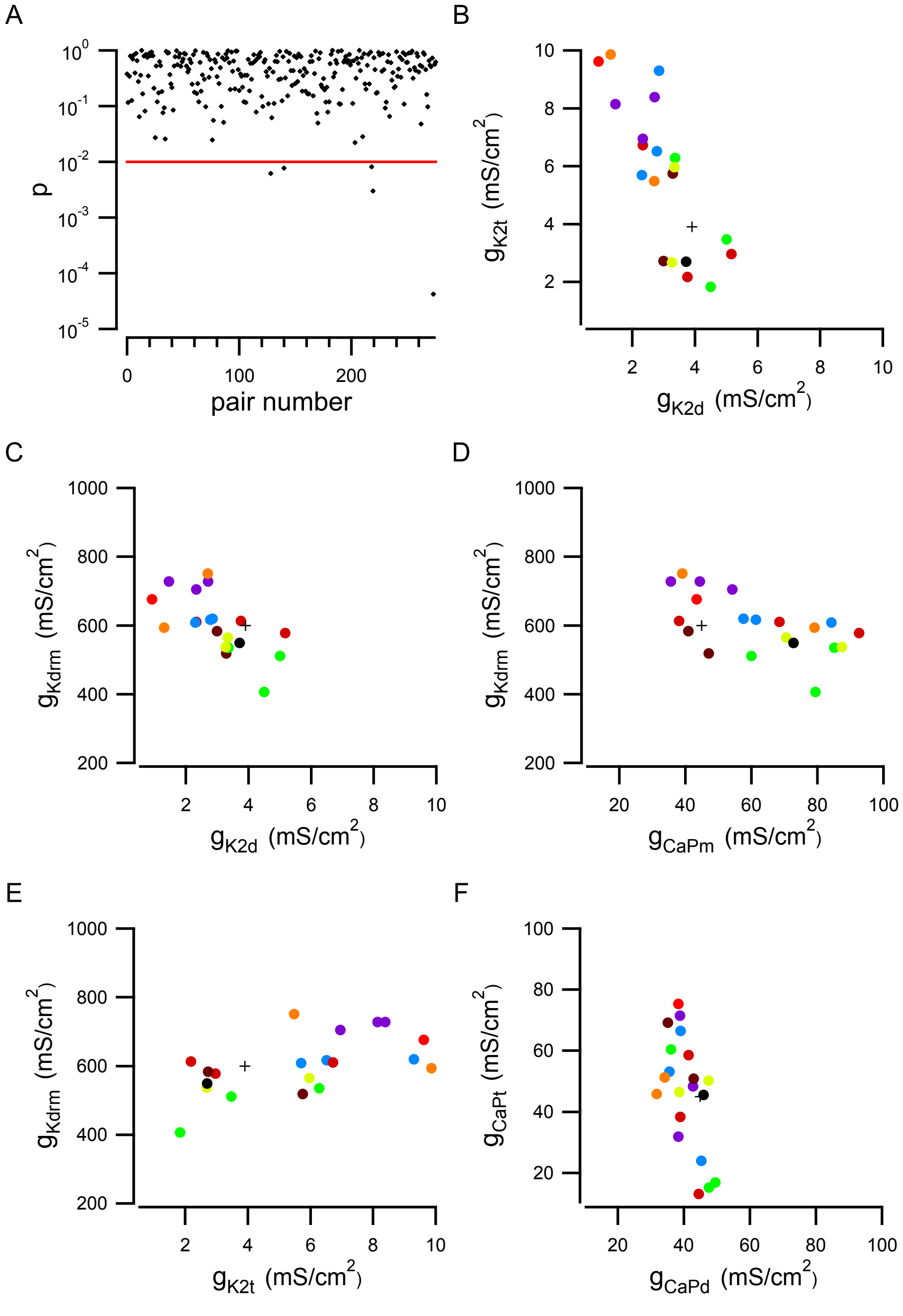

A
10^0
10^{-1}
10^{-2}
10^{-3}
10^{-4}
10^{-5}
p
0
100
200
pair number
B
g_{K2t} (mS/cm^2)
g_{K2d} (mS/cm^2)
C
g_{Kdrm} (mS/cm^2)
g_{K2d} (mS/cm^2)
D
g_{Kdrm} (mS/cm^2)
g_{CaPm} (mS/cm^2)
E
g_{Kdrm} (mS/cm^2)
g_{K2t} (mS/cm^2)
F
g_{CaPt} (mS/cm^2)
g_{CaPd} (mS/cm^2)
