# Supplementary material for: Complex Parameter Landscape for a Complex Neuron Model
Source: PLoS Comput Biol. 2006 Jul 21;2(7):e94. doi: 10.1371/journal.pcbi.0020094 (PMC1513272; doi:10.1371/journal.pcbi.0020094)
Supplement: Supplementary file 6 [file pcbi.0020094.sg006.pdf]

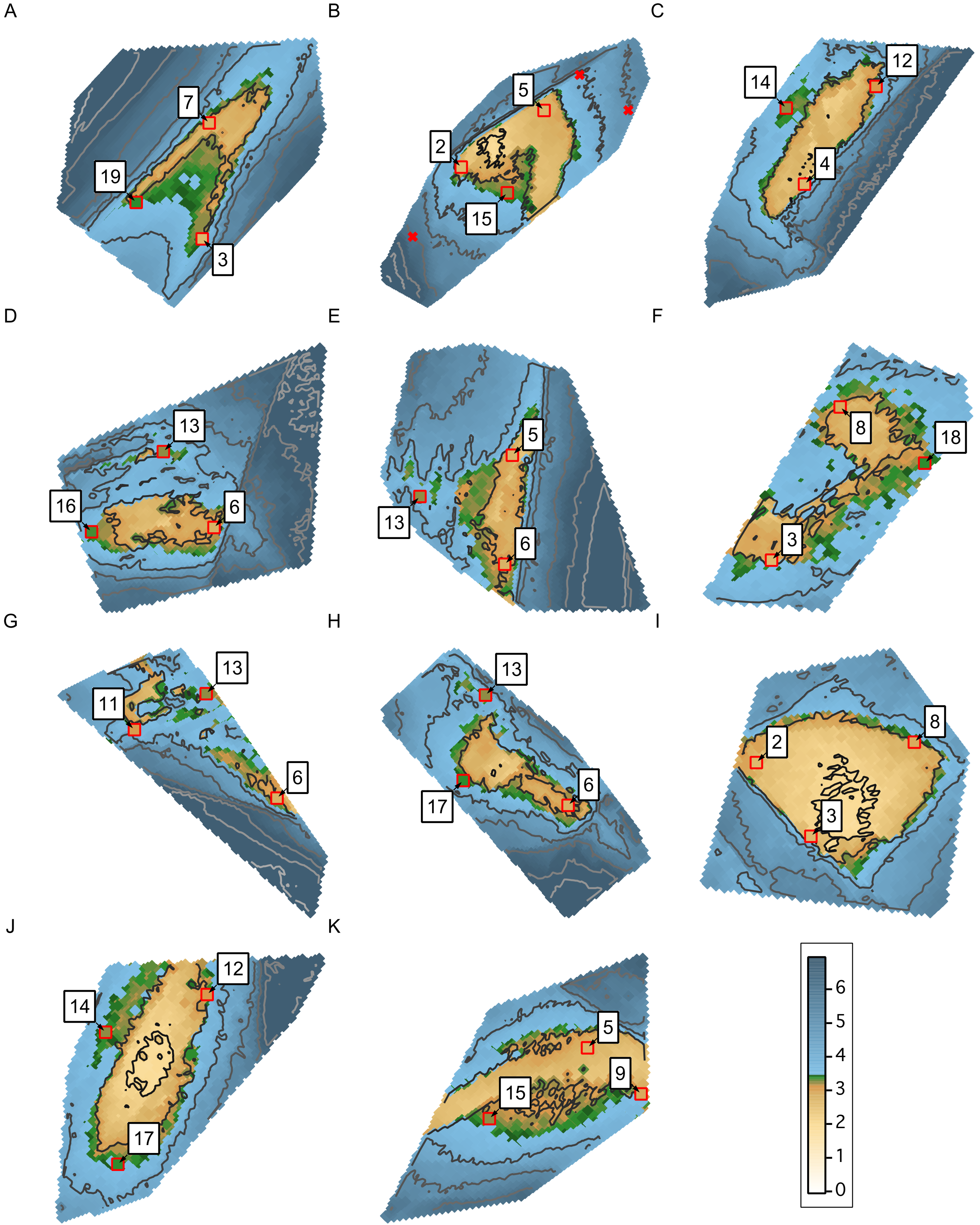

A
7
19
3
B
5
2
15
C
14
12
4
D
13
16
6
E
5
13
6
F
8
18
3
G
13
11
6
H
13
6
17
I
8
2
3
J
14
12
17
K
5
9
15
6
5
4
3
2
1
0
