# Supplementary material for: Complex Parameter Landscape for a Complex Neuron Model
Source: PLoS Comput Biol. 2006 Jul 21;2(7):e94. doi: 10.1371/journal.pcbi.0020094 (PMC1513272; doi:10.1371/journal.pcbi.0020094)
Supplement: Supplementary file 7 [file pcbi.0020094.sg007.pdf]

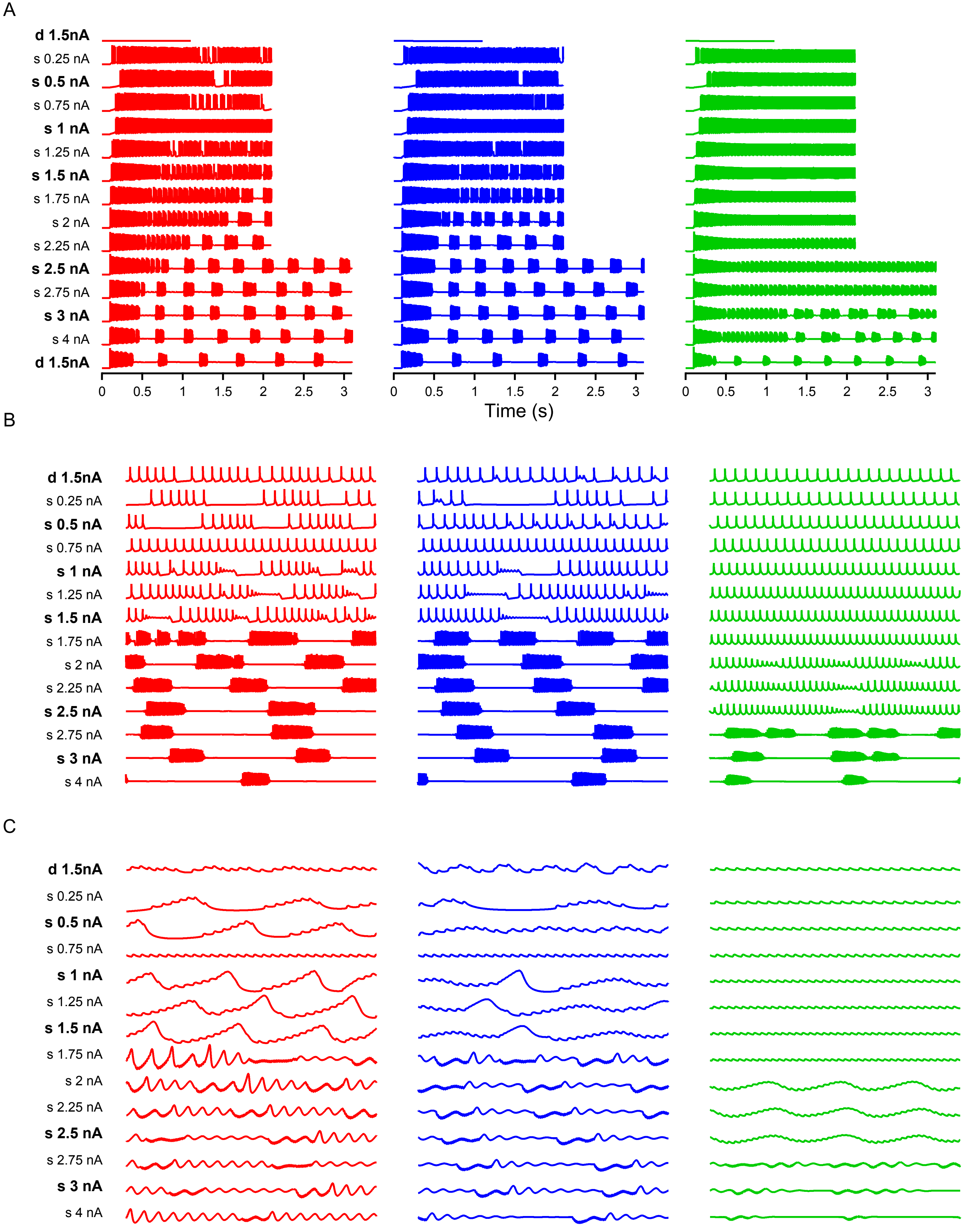

A
d 1.5nA
s 0.25 nA
s 0.5 nA
s 0.75 nA
s 1 nA
s 1.25 nA
s 1.5 nA
s 1.75 nA
s 2 nA
s 2.25 nA
s 2.5 nA
s 2.75 nA
s 3 nA
s 4 nA
d 1.5nA
0 0.5 1 1.5 2 2.5 3
Time (s)
B
d 1.5nA
s 0.25 nA
s 0.5 nA
s 0.75 nA
s 1 nA
s 1.25 nA
s 1.5 nA
s 1.75 nA
s 2 nA
s 2.25 nA
s 2.5 nA
s 2.75 nA
s 3 nA
s 4 nA
C
d 1.5nA
s 0.25 nA
s 0.5 nA
s 0.75 nA
s 1 nA
s 1.25 nA
s 1.5 nA
s 1.75 nA
s 2 nA
s 2.25 nA
s 2.5 nA
s 2.75 nA
s 3 nA
s 4 nA
