# Supplementary material for: Complex Parameter Landscape for a Complex Neuron Model
Source: PLoS Comput Biol. 2006 Jul 21;2(7):e94. doi: 10.1371/journal.pcbi.0020094 (PMC1513272; doi:10.1371/journal.pcbi.0020094)
Supplement: Supplementary file 8 [file pcbi.0020094.sd001.doc]

**Supplementary material 1**

**Fine tuning of the search method**

We have tried several variations of our search method. First, it was possible to modify some characteristics of the ES itself. We have increased the population size or changed the recombination scheme without noting any improvement. We also tested 'comma strategies' instead of 'plus strategies' (see Methods), meaning that the individuals of generation N+1 were selected among the children of the generation N only and not among the parents plus children. In our case, running a comma strategy with a population of 100 individuals producing 1000 children did not significantly change the population fitness after 12 generations.

Second, we have tested various modifications of the fitness function (Eq. 2), either by giving different weights *w_klm_* or by changing the number of *k*, *l* and *m*, i.e. changing the number of current amplitudes or excluding dendritic recordings or transitory periods. To summarize the results, it was possible to get good individuals with different fitness functions (1 dendrite instead of 2, 15 amplitudes instead of 7, etc.) but the main features of the data must be present in the fitness function, otherwise they are never recovered. For example, one cannot expect good electrical activity in the dendrites if there is not at least 1 dendritic recording. Similar conclusions were made by Keren *et al.* [8] on the number of useful dendritic recordings.

Last, it is possible to modify the resolution of the fitness function (see Eq. 1 of Methods). The size of the matrix can be changed, matrix cells can have different sizes (for example with higher resolution around subthreshold activity), the model matrix can be shifted so that the most populated points of both matrices always coincide, gaussian noise can be added to the data and so on. None of these modifications have led to significant changes in the quality of the results or in the speed with which solutions are found.
