# Supplementary material for: Complex Parameter Landscape for a Complex Neuron Model
Source: PLoS Comput Biol. 2006 Jul 21;2(7):e94. doi: 10.1371/journal.pcbi.0020094 (PMC1513272; doi:10.1371/journal.pcbi.0020094)
Supplement: Supplementary file 9 [file pcbi.0020094.sd002.doc]

**Supplementary material 2**

**Accuracy of the models**

The phase-plane analysis has previously been used by others to tune model parameters [9] as well as to characterize spike waveforms [21]. If our 20 selected models reproduce well the macroscopic features of the data (see Supp. Fig. 1), it might be useful to demonstrate how well they behave if classic measurements of spike and burst features are used. To do so, we have analyzed the electrophysiological traces of data and models in the spiking and bursting regimes.

With a very low injected current in the soma (0.25 nA), all the models were regularly spiking. In the stable period, i.e. after 1 s of recording, we measured several spike features. As in [17], the beginning of a spike is triggered when dV/dt > 100 mV/ms and a spike ends when the membrane voltage is back to its value at spike's start. Spike's peak and after-hyperpolaring potential (AHP) are defined when dV/dt = 0. It is then possible to define the spike duration, the time between the start and the peak of the spikes, the duration of AHPs, the full width at half maximum of the spikes, the inter-spike interval (ISI), the spike area, the spike amplitude, the AHP amplitude, the potential at which spikes start, the potential at half maximum of the spikes, the potential of the spike peaks and the AHPs. Comparisons of data and models for all these values are shown in Supplementary Figure 2. They all show very good agreement and the differences between data and models always remain in the range of variability observed in real electrophysiological data (typically of a couple of mV for the spike height and a few tenths of ms for the spike width [22]); this variability is lacking in our ‘data’ as it was produced by a deterministic model.

With higher injected currents in the soma (3 nA), all the models showed bursting behavior with long bursts (see typical traces on Fig. 2C). The same measures as above were performed on the spikes and are displayed in Supplementary Figure 3. All the models reproduce well not only the mean of these measures but also their sdv, which reflects the variability of spike shapes inside a burst. Bursts are defined when one ISI is larger then 120% of the mean ISI. In Supplementary Figure 4, the inter-burst interval (IBI), the duration of bursts, the number of spikes per burst and the ISI inside bursts are shown for data and models. Only individuals number 10 and 11 show significant deviation with the data. For the individual 10, this is due to a long transitory period which ends about 1.5 s after the beginning of experiment. So small bursts in the period 1-1.5 s are averaged with long bursts recorded after 1.5 s. For the individual 11, the envelope of some bursts shows two or three humps instead of one and the 120% ISI limit therefore defines more bursts than in data.

All in all, these comparisons exhibit very good agreement between data and models for detailed analysis of quantitative features of the spiking and demonstrate that the (V,dV/dt) matrix is an efficient input for fitness functions based on electrophysiological traces.

21. Naundorf B, Wolf F and Volgushev M (2006). Unique features of action potential initiation in cortical neurons. Nature 440: 1060-1063.

22. de Polavieja GG, Harsch A, Kleppe I, Robinson HPC and Juusola M (2005). Stimulus history reliably shapes action potential waveform of cortical neurons. J. Neurosci. 25: 5657-5665.
